# Supplementary material for: ProteinShader: illustrative rendering of macromolecules
Source: BMC Struct Biol. 2009 Mar 30;9:19. doi: 10.1186/1472-6807-9-19 (PMC2672931; doi:10.1186/1472-6807-9-19)
Supplement: Additional file 1 — ProteinShader program without source code. This compressed file contains the complete ProteinShader program including associated libraries, but no source code. A README.txt file gives an overview of the ProteinShader distribution, and the index.html file in the help subdirectory has directions on getting started with the program as well as a set of tutorials. [file 1472-6807-9-19-S1.zip › ProteinShader-beta-0_9_4-binary/help/api/org/proteinshader/graphics/shader/class-use/ShaderManager.html]

Uses of Class org.proteinshader.graphics.shader.ShaderManager (ProteinShader API)


|  |  |  |
| --- | --- | --- |
| **Overview** \| **Package** \| **Class** \| **Use** \| **Tree** \| **Deprecated** \| **Index** \| **Help** | |  |
| PREV   NEXT | **FRAMES**    **NO FRAMES**     **All Classes** |  |


---


## **Uses of Class org.proteinshader.graphics.shader.ShaderManager**

| Packages that use ShaderManager | |
| --- | --- |
| **org.proteinshader.graphics** | Holds the drawing classes: Ribbon, Tube, FrenetFrames, Sphere, and Cylinder. |
| **org.proteinshader.graphics.displaylists** | Holds the classes needed to manage OpenGL display lists, which are used to cache reusable geometry for spheres, cylinders, ribbon segments, and tube segments. |

Uses of ShaderManager in org.proteinshader.graphics

| Methods in org.proteinshader.graphics with parameters of type ShaderManager | |
| --- | --- |
| `void` | `FrenetFrames.setShaders(ShaderManager shaders)`             The ShaderManager is only needed because of an apparent bug on some Mac OS X machines where material color can only be set once after a custom shader is set, so to draw Frenet frames with more than one color, the shaders must be reset each time. |

Constructors in org.proteinshader.graphics with parameters of type ShaderManager

`Silhouette(ShaderManager shaders)`
  
          Constructs a Silhouette object.

Uses of ShaderManager in org.proteinshader.graphics.displaylists

| Methods in org.proteinshader.graphics.displaylists with parameters of type ShaderManager | |
| --- | --- |
| `void` | `SegmentReferences.setFrenetFramesShaders(ShaderManager shaders)`             Makes the ShaderManager object available for use in the FrenetFrames object. |

---


|  |  |  |
| --- | --- | --- |
| **Overview** \| **Package** \| **Class** \| **Use** \| **Tree** \| **Deprecated** \| **Index** \| **Help** | |  |
| PREV   NEXT | **FRAMES**    **NO FRAMES**     **All Classes** |  |


---

# *Copyright © 2007-2008*
